# A Rare Case of Myoepithelioma in the Sphenoid Sinus

**DOI:** 10.1002/ccr3.71652

**Published:** 2025-12-07

**Authors:** Xing‐hao Wang, Ying Zhou

**Affiliations:** ^1^ School of Medical Imaging Chengdu Medical College Chengdu China; ^2^ Department of Radiology Mianyang Central Hospital Mianyang China

**Keywords:** head and neck neoplasms, magnetic resonance imaging, myoepithelioma, sphenoid sinus, tomography

## Abstract

A 72‐year‐old male was admitted to the hospital with 3 months of vision loss and 1 month of aggravation. Computed tomography and magnetic resonance imaging examinations revealed a tumor in the sphenoid region, which was treated with surgical resection. Based on the pathology and immunohistochemistry findings, the tumor was diagnosed as a myoepithelial tumor. The patient was in good condition after surgery. Myoepithelial tumors are rare, with an even rarer occurrence in the sphenoid sinuses, where such tumors are particularly challenging to diagnose preoperatively. Diagnosis relies on postoperative pathology and immunohistochemistry. Surgery remains the primary treatment approach.

## Key Clinical Message

Myoepithelial tumors within the paranasal sinuses are rare, with an even rarer occurrence in the sphenoid sinuses and are challenging to diagnose on preoperative imaging. Clinicians should consider this diagnosis in patients presenting with similar symptoms in the paranasal sinuses.

## 1 Introduction

Myoepithelial tumors are uncommon tumors with no specific predilection for age or sex. Myoepithelial tumors of the head and neck are predominantly found in the salivary glands, whereas those located in the sinuses are rare [1, 2]. And to our knowledge, there are no reports in the literature on myoepitheliomas located in the sphenoid sinus. The most common lesions in the sphenoid sinus are inflammatory lesions. Next are the mucocoeles. Tumors are the least common. Benign tumors or tumor‐like lesions include osteomas, giant cell tumors, schwannomas, and fibromas. Malignant tumors are less common and typically include adenocarcinomas, squamous cell carcinomas, epidermoid carcinomas, and metastatic lesions [3]. In imaging examinations, it typically presents as a mass in the sphenoid sinus. An excessively large mass may obstruct the nasal meatus, causing nasal congestion. Malignant tumors can also cause destruction of surrounding bone tissue and invade nearby blood vessels and nerves, leading to complications such as decreased visual acuity and facial numbness. We report a case of an older male patient with a lesion in the sphenoid sinus who was admitted to the hospital with decreased visual acuity. Preoperative computed tomography (CT) and magnetic resonance imaging (MRI) indicated a mass in the sphenoid sinus, and postoperative pathology confirmed the diagnosis. Myoepitheliomas lack distinctive imaging, making preoperative diagnosis challenging. Consequently, diagnosis relies on postoperative pathology and immunohistochemistry. Surgery is currently an effective treatment modality; however, the efficacy of radiotherapy and chemotherapy remains unclear.

## 2 Case Presentation

A 72‐year‐old male presented with a history of progressive vision loss without an apparent cause 3 months before admission. No associated diplopia, limitation of eye movement, rotation, eyelid ptosis, dizziness, or headache was observed. Approximately 1 month later, the patient underwent cataract surgery at another hospital, after which his vision further deteriorated, resulting in blurred vision in the left eye and blindness in the right eye. This was accompanied by distal numbness in the right limb. Cranial CT revealed partial bone loss at the right anterior skull base and significant soft tissue thickening, raising suspicion of a tumor. Personal history and family history were unremarkable.

His physical examination showed the left eyeball is inwardly retracted and misaligned, with a pupil diameter of approximately 4.0 mm, and the light reflex is absent. The right eye was blind. Frontal facial pain and touch sensation were symmetric. The bilateral finger‐to‐nose test was steady and accurate, but the heel–knee‐tibia test was uncooperative.

## 3 Investigations and Treatment

**FIGURE 1:**
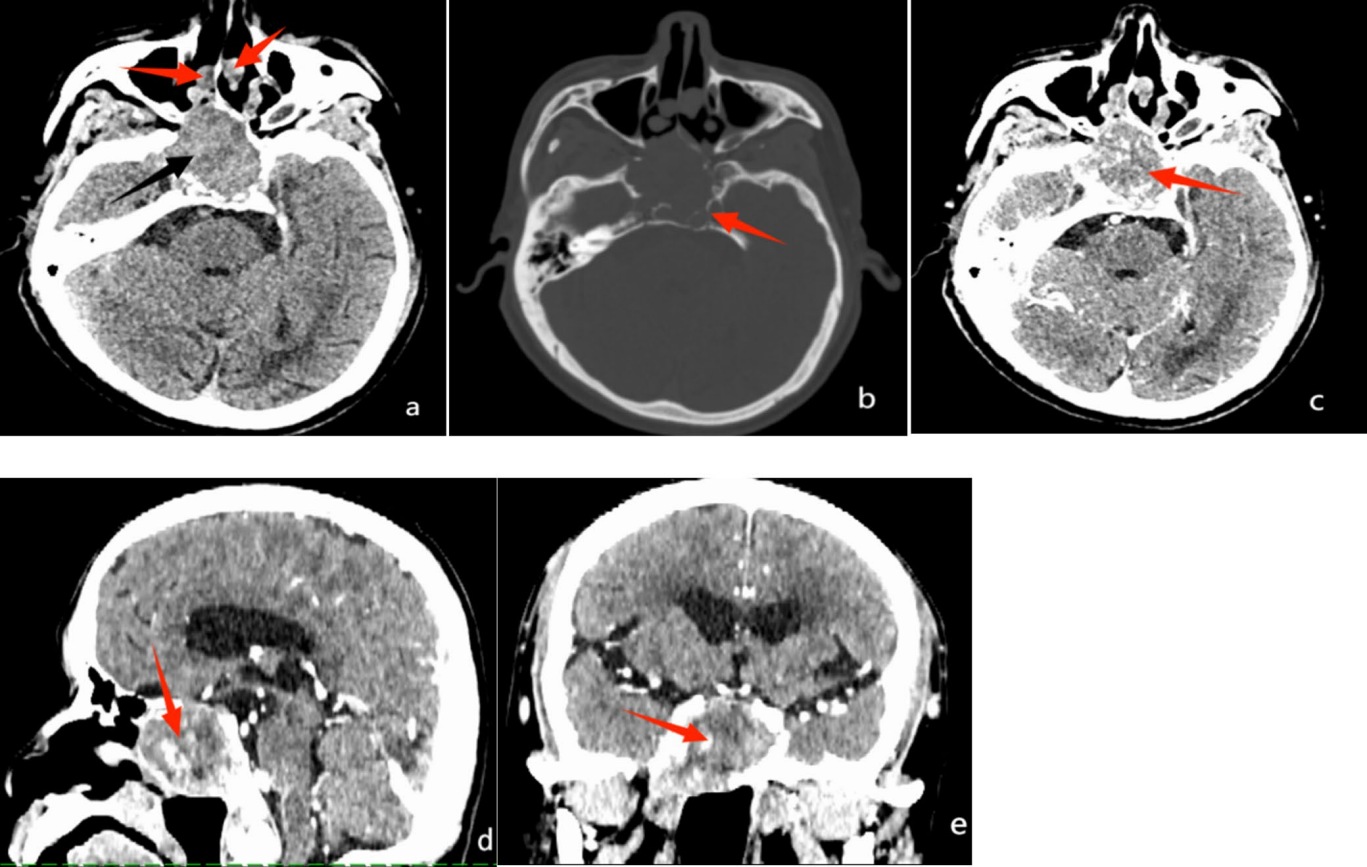
(a) Plain computed tomography (CT) scan showing occupancy in the sphenoid sinus area (black arrow) and several nodules in the nasal cavity (red arrows). (b) Plain CT bone window shows bone resorption and destruction of the saddle base and occipital slope (red arrow) around the lesion. (c–e) Transverse, sagittal, and coronal enhancement images, respectively, illustrating the inhomogeneous enhancement of the mass (red arrow).

**FIGURE 2:**
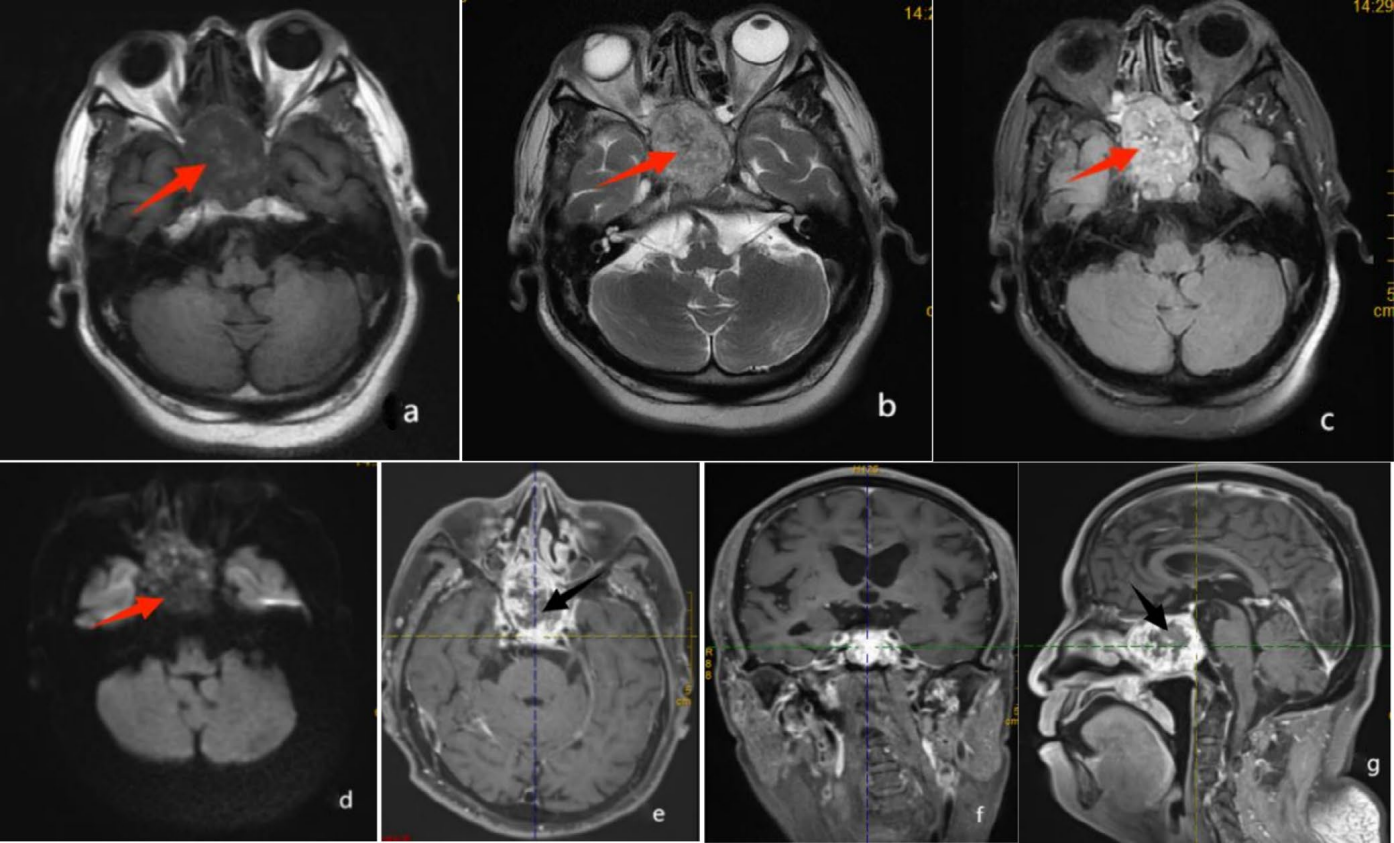
Plain and enhanced magnetic resonance images of the patient with a mass shadow in the sphenoid sinus region. (a) T1‐weighted imaging showing an equal and slightly low signal, with a few slightly high‐signal shadows in the center (red arrow). (b) T2‐weighted imaging and (c) T2FLAIR imaging showing a mixed, slightly high signal (red arrow). (d) Diffusion‐weighted imaging showing the mass as mostly isointense, with a few localized spots of slightly high signal (red arrow). (e–g) Enhancement images showing obvious uneven enhancement, with a patchy, non‐enhanced area in the center (black arrow), and the mass is not clearly demarcated from the pituitary gland.

The plain CT (Figure 1) revealed an irregular mass in the sphenoid sinus area, with marked uneven enhancement following contrast administration. The mass protruded forward behind the right nasal passages and extended upward, where it was indistinctly demarcated from the pituitary gland. The optic nerves at both orbital apices were poorly differentiated from the mass. Additionally, the saddle base, occipital slopes, inner and outer lateral walls of the right lateral sphenoid process, and the right median cranial recess had been damaged and resorbed. The palate showed several nodules with significant enhancement, along with localized destruction of the hard palate bone. MRI (Figure 2) showed the lesion as an iso‐low signal on T1‐weighted imaging (T1WI), with a patchy and slightly high signal in the center. The lesion appeared mixed, with slightly high signals on T2WI and T2 fluid‐attenuated inversion recovery (T2FLAIR). Diffusion‐weighted imaging revealed a mixed, patchy, and slightly high signal. The lesion exhibited significant uneven enhancement and patchy and non‐enhanced areas in the center.

The patient underwent an endoscopic approach to the sphenoid sinus tumor resection. Intraoperatively, bilateral middle turbinates were found to be enlarged, had congested mucosa, and were subsequently excised. A tumor was detected in the sphenoid sinus, and the mass filled the entire sphenoid sinus cavity, with some of the bone of the sphenoid sinus wall destroyed, although the dura mater of the saddle base remained intact. The mass compressed the optic nerve, displacing it to the right and upward. Additionally, partial inflation was observed in the posterior nostrils in the palate. Upon mucosal incision, the mass appeared grayish‐red, had a soft texture, a rich blood supply, and contained necrotic tissue. The surgical procedure was successful, and no intra‐op events occurred.

**FIGURE 3:**
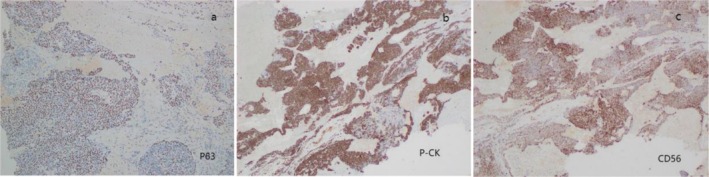
(a) P63 (punctuation +), (b) P‐CK (+), (c) CD56 (+).

**FIGURE 4:**
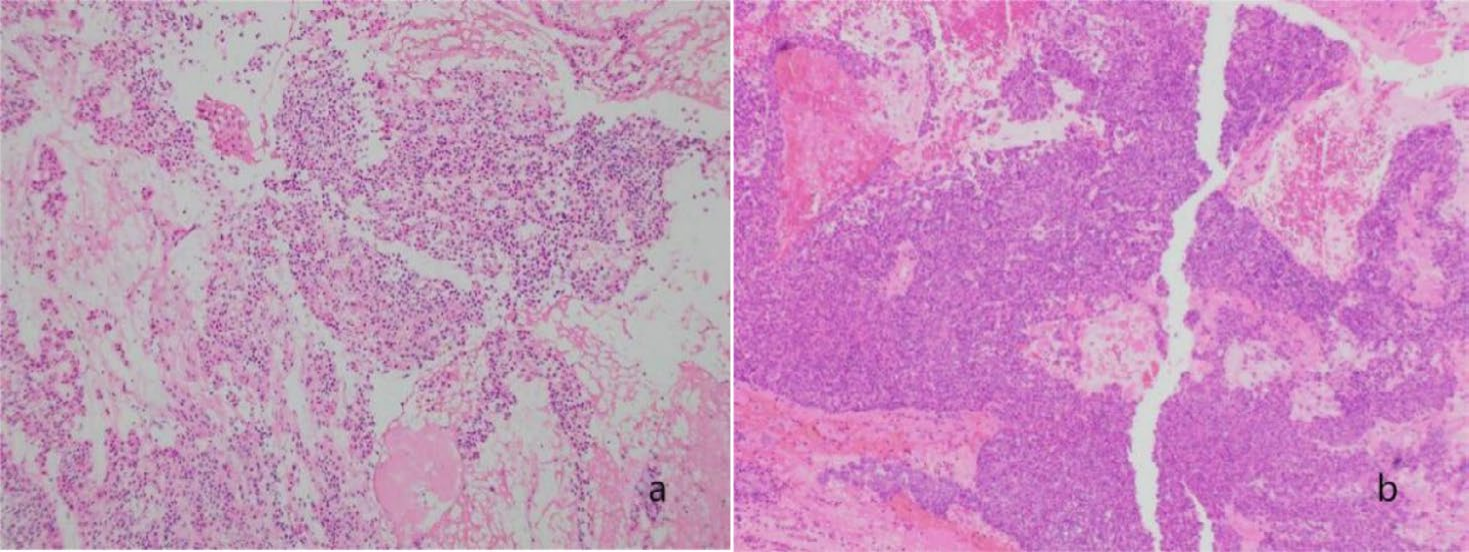
(a–b) Tumor cells were distributed in nested clusters with eosinophilic cytoplasm and medium‐sized nuclei.

The final diagnosis was myoepithelial tumor, and immunohistochemistry (Figure 3) and HE staining (Figure 4) suggest that the tumor may be malignant.

## 4 Results and Follow‐Up

**FIGURE 5:**
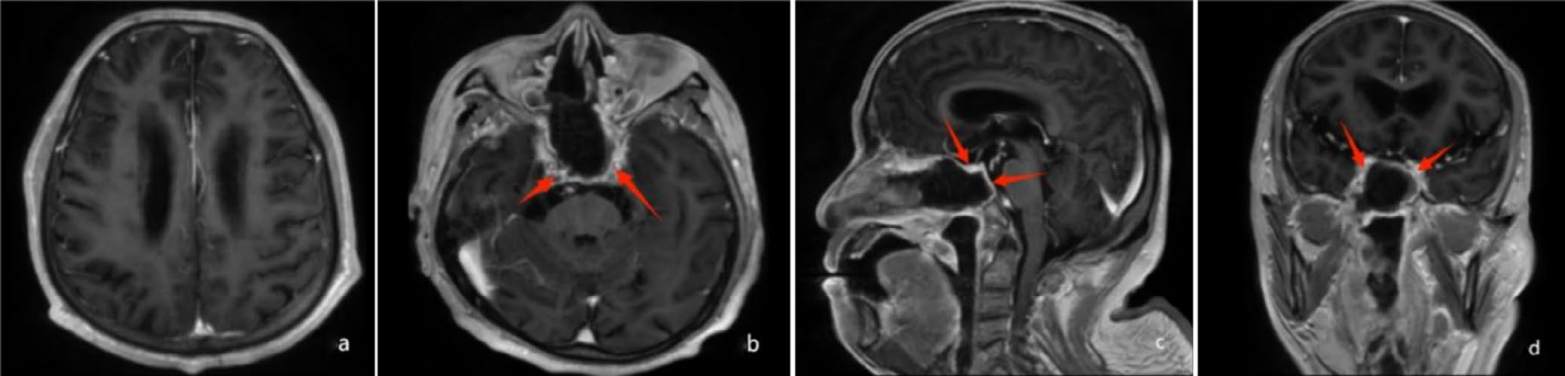
(a) Enhancement images showing no abnormalities in the brain parenchyma after surgery. (b–d) Enhancement images showing that the margin of the operative area in the sellar region was enhanced.

Postoperative follow‐up MRI (Figure 5) at 1 month showed that the margin of the operative area in the sellar region was enhanced. Following surgery, the patient retains only light perception in the right eye, while vision in the left eye has improved compared to before. The patient recovered well and was discharged after surgery.

## 5 Discussion

Myoepithelial tumors are very rare epithelial tumors, with approximately 15% occurring in the head and neck region [4]. Approximately 90% of myoepitheliomas are benign [5], but they can transform into myoepithelial carcinoma, though this occurs in < 10% of cases. Most myoepithelial carcinomas located in the head predominantly occur in salivary glands, and the parotid glands is the majority (30%–80%) [6]. Reports of myoepithelial tumors occurring in the sinuses are rare, and most are located in the maxillary sinus or, occasionally, in the ethmoid sinus [7]. Most cases located in the nasal and paranasal sinuses present clinically with nasal obstruction and recurrent epistaxis [8]. Cases located in the ethmoid sinus present with eye pain, diplopia, proptosis, and tearing on the affected side [7], which may be related to orbital tumor invasion. The pathogenesis of myoepithelial carcinoma remains controversial, with some studies suggesting that it may occur independently, whereas others suggest a malignant transformation from pleomorphic adenoma or a benign myoepithelial tumor [9].

In this case, this patient was admitted to the hospital with decreased and blurred vision, and the myoepithelioma was located in the sphenoid sinus, a site not previously reported in the literature. CT and MRI examinations revealed occupancy of the sphenoid saddle region, which was later confirmed as a myoepithelioma through immunohistochemistry. However, the possibility of myoepithelial carcinoma could not be ruled out due to imaging findings, including evident bone destruction around the tumor (saddle base, occipital slope, inner and outer lateral panels of the right sphenoid process, and the right midcranial recess), and several enhanced nodules in the palate. Localized bone destruction in the hard palate was also observed, and S100 (scattered +) was observed by immunohistochemistry.

For myoepithelial tumors located in the sinus, CT and MRI examinations typically show a soft tissue mass with uneven enhancement. If it is accompanied by destruction and invasion of the surrounding soft tissue, or neoplastic soft tissue nodules are seen in the surrounding tissue structure (as in this case), then there is a possibility of malignancy. However, this is not unique to myoepithelial carcinomas and may not be accompanied by peripheral tissue destruction. Identifying benign and malignant tumors requires a combination of cytological patterns, including cytological heterogeneity, cellular pleomorphism, mitotic activity, necrosis, and infiltrative growth.

Immunohistochemistry is crucial in distinguishing myoepithelial carcinomas from other tumors, with markers like S100, actin, or cytokeratin 14 being commonly expressed [10]. According to the latest literature, high‐grade myoepithelial carcinoma may be related to INI‐1 loss [11]. Surgery remains the primary treatment for myoepithelial tumors. Although radiation therapy and chemotherapy have also been used to treat malignant cases, there is no consensus on the need for postoperative adjuvant therapy given the disease's unclear prognosis. Some studies suggest that radiotherapy or concurrent radiochemotherapy may be effective for treating local recurrence and distant metastasis [12, 13]. However, another report indicated a lack of efficacy for adjuvant therapy in both local and distant recurrence [14], with the overall prognosis for malignant myoepitheliomas being poor.

## 6 Conclusion

Myoepithelial tumors within the paranasal sinuses are rare and challenging to diagnose on preoperative imaging, with difficulties in distinguishing between benign and malignant forms. Clinicians should consider this diagnosis in patients presenting with similar symptoms in the paranasal sinuses. Confirmatory diagnosis relies on pathology and immunohistochemistry. Surgery is the main treatment approach, and the role of radiotherapy remains uncertain and should be tailored to the patient's specific condition.

## Author Contributions

**Xing‐hao Wang:** resources, visualization, writing – original draft, writing – review and editing. **Ying Zhou:** resources, writing – review and editing.

## Funding

The authors have nothing to report.

## Ethics Statement

This study was approved by the Ethics Committee of the Mianyang Central Hospital.

## Consent

Written informed consent was obtained from the patient to publish this report in accordance with the journal's patient consent policy.

## Conflicts of Interest

The authors declare no conflicts of interest.

## Data Availability

The data that support the findings of this study are available upon request from the corresponding author.
